# RETRACTION: Pard3 Suppresses Glioma Invasion by Regulating RhoA Through Atypical Protein Kinase C/NF‐κB Signaling

**DOI:** 10.1002/cam4.70408

**Published:** 2024-11-15

## Abstract

**RETRACTION:** J. Li, H. Xu, Q. Wang, P. Fu, T. Huang, O. Anas, H. Zhao and N. Xiong, “Pard3 Suppresses Glioma Invasion by Regulating RhoA Through Atypical Protein Kinase C/NF‐κB Signaling,” *Cancer Medicine* 8, no. 5 (2019): 2288–2302, https://doi.org/10.1002/cam4.2063.

The above article, published online on 07 March 2019 in Wiley Online Library (wileyonlinelibrary.com), has been retracted by agreement between the journal Editor‐in‐Chief, Stephen Tait; and John Wiley & Sons Ltd. The retraction has been agreed due to several instances of image overlaps observed in Figures 7C, 2F, 2G, 4F, 4G, 2D, 3D, 4D, 2E, 3E and 4E. Furthermore, elements from Figures 2F, 2G and 4G were found published in an article elsewhere in the same year by a different author group. Additionally, elements from Figures 2E and 3E were also published in another article in the same year by some of the same authors. The authors responded to the concerns and provided some data. However, their explanation and data provided were insufficient. Due to the nature and extent of the duplications, the editors consider the results and conclusion of this article to be invalid. The authors disagree with the retraction.

**RETRACTION:** J. Li, H. Xu, Q. Wang, P. Fu, T. Huang, O. Anas, H. Zhao and N. Xiong, “Pard3 Suppresses Glioma Invasion by Regulating RhoA Through Atypical Protein Kinase C/NF‐κB Signaling,” *Cancer Medicine* 8, no. 5 (2019): 2288–2302, https://doi.org/10.1002/cam4.2063.

The above article, published online on 07 March 2019 in Wiley Online Library (wileyonlinelibrary.com), has been retracted by agreement between the journal Editor‐in‐Chief, Stephen Tait; and John Wiley & Sons Ltd. The retraction has been agreed due to several instances of image overlaps observed in Figures 7C, 2F, 2G, 4F, 4G, 2D, 3D, 4D, 2E, 3E and 4E. Furthermore, elements from Figures 2F, 2G and 4G were found published in an article elsewhere in the same year by a different author group. Additionally, elements from Figures 2E and 3E were also published in another article in the same year by some of the same authors. The authors responded to the concerns and provided some data. However, their explanation and data provided were insufficient. Due to the nature and extent of the duplications, the editors consider the results and conclusion of this article to be invalid. The authors disagree with the retraction.

